# Ethyl 3-[7-(*N*-acetyl-4-meth­oxy­benzene­sulfonamido)-3-chloro-2*H*-indazol-2-yl]propionate

**DOI:** 10.1107/S1600536814003183

**Published:** 2014-02-15

**Authors:** Assoman Kouakou, El Mostapha Rakib, Najat Abbassi, Mohamed Saadi, Lahcen El Ammari

**Affiliations:** a Laboratoire de Chimie Organique et Analytique, Université Sultan Moulay Slimane, Faculté des Sciences et Techniques, Béni-Mellal, BP 523, Morocco; b Laboratoire de Chimie du Solide Appliquée, Faculté des Sciences, Université Mohammed V-Agdal, Avenue Ibn Battouta, BP. 1014, Rabat, Morocco

## Abstract

In the title compound, C_21_H_22_ClN_3_O_6_S, the fused five- and six-membered ring rings are almost perpendicular to the planes through the atoms forming the acetyl and the propionic ester groups, as indicated by the dihedral angles of 80.3 (2) and 88.3 (7)°, respectively. The dihedral angle between the indazole system and the 4-meth­oxy­benzene­sulfonyl group is 13.76 (6)°. The carbonyl O atom is split over two positions in a 0.60 (5):0.40 (5) ratio. In the crystal, mol­ecules are linked by C—H⋯O and C—H⋯N inter­actions into a three-dimensional network.

## Related literature   

For the biological activity of sulfonamides, see: Lohou *et al.* (2012 ▶); Salerno *et al.* (2012 ▶); Kaltenbach *et al.* (2003 ▶); Thangadurai *et al.* (2012 ▶); Abbassi *et al.* (2012 ▶). For similar compounds, see: Abbassi *et al.* (2013 ▶); Chicha *et al.* (2013 ▶).
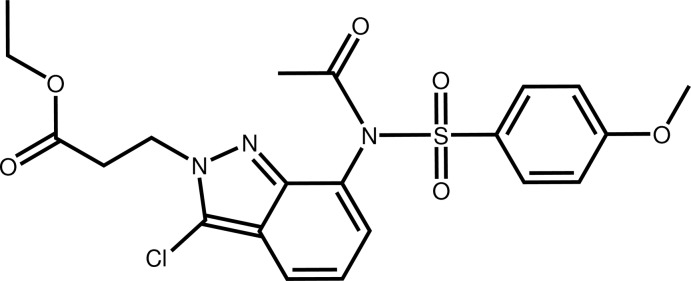


## Experimental   

### 

#### Crystal data  

C_21_H_22_ClN_3_O_6_S
*M* *_r_* = 479.93
Triclinic, 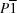

*a* = 9.1442 (3) Å
*b* = 9.4376 (4) Å
*c* = 14.0931 (6) Å
α = 108.262 (2)°
β = 96.017 (2)°
γ = 103.313 (2)°
*V* = 1103.12 (8) Å^3^
*Z* = 2
Mo *K*α radiation
μ = 0.31 mm^−1^
*T* = 296 K
0.38 × 0.32 × 0.27 mm

#### Data collection  

Bruker X8 APEX diffractometer
Absorption correction: multi-scan (*SADABS*; Bruker, 2009 ▶) *T*_min_ = 0.700, *T*_max_ = 0.746
26786 measured reflections
6725 independent reflections
4687 reflections with *I* > 2σ(*I*)
*R*_int_ = 0.030

#### Refinement  

*R*[*F* ^2^ > 2σ(*F* ^2^)] = 0.043
*wR*(*F* ^2^) = 0.123
*S* = 1.02
6725 reflections
299 parameters
H-atom parameters constrained
Δρ_max_ = 0.30 e Å^−3^
Δρ_min_ = −0.34 e Å^−3^

### 

Data collection: *APEX2* (Bruker, 2009 ▶); cell refinement: *SAINT* (Bruker, 2009 ▶); data reduction: *SAINT*; program(s) used to solve structure: *SHELXS97* (Sheldrick, 2008 ▶); program(s) used to refine structure: *SHELXL97* (Sheldrick, 2008 ▶); molecular graphics: *ORTEP-3 for Windows* (Farrugia, 2012 ▶); software used to prepare material for publication: *PLATON* (Spek, 2009 ▶) and *publCIF* (Westrip, 2010 ▶).

## Supplementary Material

Crystal structure: contains datablock(s) I. DOI: 10.1107/S1600536814003183/tk5294sup1.cif

Structure factors: contains datablock(s) I. DOI: 10.1107/S1600536814003183/tk5294Isup2.hkl

Supporting information file. DOI: 10.1107/S1600536814003183/tk5294Isup3.cml

CCDC reference: 986487

Additional supporting information:  crystallographic information; 3D view; checkCIF report

## Figures and Tables

**Table 1:** Hydrogen-bond geometry (Å, °)

*D*—H⋯*A*	*D*—H	H⋯*A*	*D*⋯*A*	*D*—H⋯*A*
C2—H2*A*⋯O4^i^	0.97	2.58	3.438 (3)	148
C21—H21*A*⋯O2*B* ^ii^	0.96	2.44	3.273 (7)	144
C21—H21*A*⋯O2*A* ^ii^	0.96	2.60	3.43 (2)	145
C2—H2*B*⋯O6^iii^	0.97	2.71	3.510 (3)	140
C14—H14*C*⋯N2^iv^	0.96	2.55	3.501 (2)	173
C14—H14*B*⋯O5^v^	0.96	2.45	3.348 (2)	155

Symmetry codes: (i) 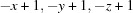; (ii) 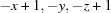; (iii) 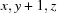; (iv) 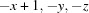; (v) 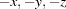.

**Fig. 1.**
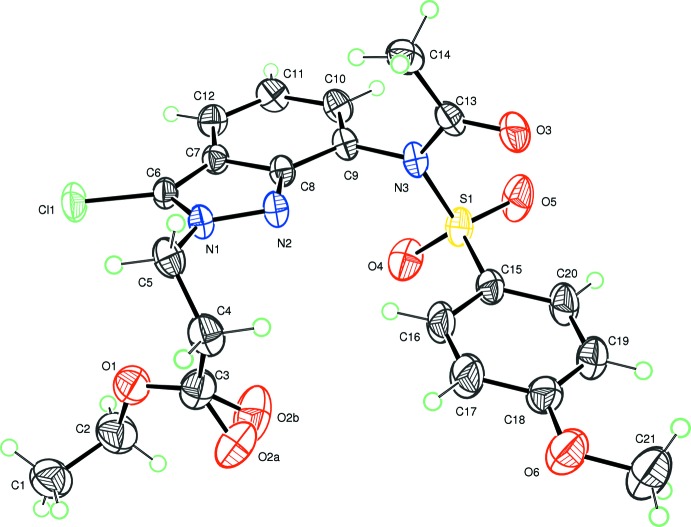
Plot of the molecule of the title compound with the atom-labelling scheme. Displacement ellipsoids are drawn at the 50% probability level.

**Fig. 2.**
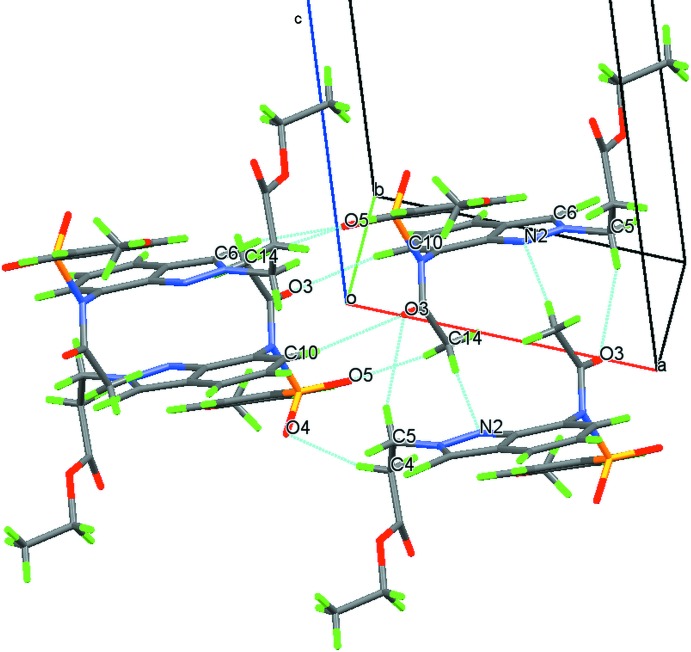
Partial crystal packing for the title compound showing hydrogen bonds as dashed lines.

## supplementary crystallographic information

### 2. Structural commentary

The indazole ring system is recognized to be a highly effective pharmacophore in medicinal chemistry as well as being the core of important nitro­gen-containing heterocycles that show a broad range of biological activities, such as nitric oxide syntheses and HIV protease inhibitors, anti-inflammatory and anti-cancer agents, and serotonin 5-HT3 receptor antagonists (Lohou *et al.*, 2012; Salerno *et al.*, 2012; Kaltenbach *et al.*, 2003; Thangadurai *et al.*, 2012; Abbassi *et al.*, 2012). The present work is a continuation of the investigation of the sulfonamide derivatives published recently by our team (Abbassi, *et al.*, 2013; Chicha, *et al.*, 2013).

The molecule of the title compound is built up from fused five- and six-membered rings linked to an acetyl-(4-meth­oxy-benzene­sulfonyl)-amino group and to a propionic ethyl ester as shown in Fig. 1. The indazole ring system makes dihedral angles of 80.3 (2) and 88.3 (7)°, with the two planes through the atoms forming the acetyl (O3C13C14) and the propionic ester (O1O2C3C4C5) groups, respectively. The plane through the 4-meth­oxy-benzene­sulfonyl group is nearly parallel to the indazole ring system, as indicated by the dihedral angle of 13.76 (6)°. In the crystal, the molecules are linked by C—H···O and C—H···N inter­actions to form a three-dimensional network (Fig. 2 and Table 1).

### 5. Synthesis and crystallization

Ethyl 3-(3-chloro-7-nitro-2*H*-indazol-2-yl)propano­ate (1.48 mmol) was added to a mixture of indium powder (850 mg, 7.43 mmol) and acetic acid (8.35 ml, 150 mmol) in ethanol (5 ml). The reaction mixture was stirred at 333 K for 6 h. After reduction, the starting material disappeared, and the solution was allowed to cool. The pH was made slightly basic (pH 7–8) by addition of 5% aqueous potassium bicarbonate before extraction with ethyl acetate. The organic phase was washed with brine and dried over magnesium sulfate. The solvent was removed to afford the amine, which was immediately dissolved in pyridine (5 ml) and then reacted with 4-meth­oxy­benzene­sulfonyl chloride (1.25 mmol) at room temperature for 24 h. After the reaction mixture was concentrated *in vacuo*, the resulting residue was purified by flash chromatography (eluted with ethyl acetate: Hexane 2:8). The title compound was recrystallized from ethanol (Yield: 44%; M.pt: 394 K).

### 6. Refinement

H atoms were located in a difference map and treated as riding with C–H = 0.96 Å, 0.97 Å and 0.93 Å for methyl-, methyl­ene- and aromatic-H, respectively, and with *U*_iso_(H) = 1.5 *U*_eq_ for methyl-H and *U*_iso_(H) = 1.2 *U*_eq_ for methyl­ene- and aromatic-H. The ester-O atom was rfined over two positions, O2A and O2B, in a 40:60 ratio.

### Crystal data

**Table:**

C_21_H_22_ClN_3_O_6_S	*Z* = 2
*M**_r_* = 479.93	*F*(000) = 500
Triclinic, *P*1	*D*_x_ = 1.445 Mg m^−^^3^
Hall symbol: -P 1	Mo *K*α radiation, λ = 0.71073 Å
*a* = 9.1442 (3) Å	Cell parameters from 6725 reflections
*b* = 9.4376 (4) Å	θ = 2.4–30.5°
*c* = 14.0931 (6) Å	µ = 0.31 mm^−^^1^
α = 108.262 (2)°	*T* = 296 K
β = 96.017 (2)°	Block, colourless
γ = 103.313 (2)°	0.38 × 0.32 × 0.27 mm
*V* = 1103.12 (8) Å^3^	

### Data collection

**Table:**

Bruker X8 APEX diffractometer	6725 independent reflections
Radiation source: fine-focus sealed tube	4687 reflections with *I* > 2σ(*I*)
Graphite monochromator	*R*_int_ = 0.030
φ and ω scans	θ_max_ = 30.5°, θ_min_ = 2.4°
Absorption correction: multi-scan (*SADABS*; Bruker, 2009)	*h* = −13→13
*T*_min_ = 0.700, *T*_max_ = 0.746	*k* = −13→12
26786 measured reflections	*l* = −19→20

### Refinement

**Table:**

Refinement on *F*^2^	Primary atom site location: structure-invariant direct methods
Least-squares matrix: full	Secondary atom site location: difference Fourier map
*R*[*F*^2^ > 2σ(*F*^2^)] = 0.043	Hydrogen site location: inferred from neighbouring sites
*wR*(*F*^2^) = 0.123	H-atom parameters constrained
*S* = 1.02	*w* = 1/[σ^2^(*F*_o_^2^) + (0.0567*P*)^2^ + 0.2297*P*] where *P* = (*F*_o_^2^ + 2*F*_c_^2^)/3
6725 reflections	(Δ/σ)_max_ < 0.001
299 parameters	Δρ_max_ = 0.30 e Å^−^^3^
0 restraints	Δρ_min_ = −0.34 e Å^−^^3^

### Special details

**Table:**

Geometry. All e.s.d.'s (except the e.s.d. in the dihedral angle between two l.s. planes) are estimated using the full covariance matrix. The cell e.s.d.'s are taken into account individually in the estimation of e.s.d.'s in distances, angles and torsion angles; correlations between e.s.d.'s in cell parameters are only used when they are defined by crystal symmetry. An approximate (isotropic) treatment of cell e.s.d.'s is used for estimating e.s.d.'s involving l.s. planes.
Refinement. Refinement of *F*^2^ against all reflections. The weighted *R*-factor *wR* and goodness of fit *S* are based on *F*^2^, conventional *R*-factors *R* are based on *F*, with *F* set to zero for negative *F*^2^. The threshold expression of *F*^2^ > σ(*F*^2^) is used only for calculating *R*-factors(gt) *etc*. and is not relevant to the choice of reflections for refinement. *R*-factors based on *F*^2^ are statistically about twice as large as those based on *F*, and *R*- factors based on all data will be even larger.

### Fractional atomic coordinates and isotropic or equivalent isotropic displacement parameters (Å^2^)

**Table:**

	*x*	*y*	*z*	*U*_iso_*/*U*_eq_	Occ. (<1)
C1	1.0486 (3)	0.8525 (3)	0.54237 (17)	0.0722 (6)	
H1A	1.0445	0.9308	0.6042	0.108*	
H1B	1.0923	0.9013	0.4975	0.108*	
H1C	1.1106	0.7903	0.5579	0.108*	
C2	0.8924 (3)	0.7528 (3)	0.49251 (17)	0.0745 (6)	
H2A	0.8474	0.7029	0.5373	0.089*	
H2B	0.8285	0.8150	0.4774	0.089*	
C3	0.8483 (2)	0.4891 (2)	0.38663 (16)	0.0538 (4)	
C4	0.8705 (2)	0.3834 (2)	0.28825 (15)	0.0522 (4)	
H4A	0.8013	0.2809	0.2726	0.063*	
H4B	0.9742	0.3743	0.2970	0.063*	
C5	0.84382 (17)	0.43617 (19)	0.19883 (13)	0.0449 (4)	
H5A	0.9164	0.5362	0.2122	0.054*	
H5B	0.8617	0.3625	0.1387	0.054*	
C6	0.64292 (16)	0.56845 (16)	0.16545 (11)	0.0358 (3)	
C7	0.48449 (16)	0.52038 (16)	0.13433 (11)	0.0345 (3)	
C8	0.44533 (15)	0.36588 (15)	0.13440 (11)	0.0327 (3)	
C9	0.28989 (16)	0.27675 (16)	0.10605 (11)	0.0355 (3)	
C10	0.18252 (17)	0.34113 (19)	0.07555 (13)	0.0435 (4)	
H10	0.0804	0.2829	0.0551	0.052*	
C11	0.2236 (2)	0.4947 (2)	0.07452 (13)	0.0477 (4)	
H11	0.1477	0.5347	0.0529	0.057*	
C12	0.37178 (19)	0.58583 (18)	0.10434 (12)	0.0429 (3)	
H12	0.3973	0.6873	0.1048	0.051*	
C13	0.25249 (17)	−0.01017 (17)	0.03603 (12)	0.0423 (3)	
C14	0.3172 (2)	0.01302 (19)	−0.05230 (13)	0.0488 (4)	
H14A	0.4034	0.1038	−0.0286	0.073*	
H14B	0.2405	0.0264	−0.0981	0.073*	
H14C	0.3494	−0.0761	−0.0872	0.073*	
C15	0.28344 (17)	0.00934 (18)	0.26812 (12)	0.0426 (3)	
C16	0.4413 (2)	0.0666 (2)	0.29788 (16)	0.0613 (5)	
H16	0.4928	0.1571	0.2887	0.074*	
C17	0.5205 (2)	−0.0119 (3)	0.34089 (19)	0.0711 (6)	
H17	0.6266	0.0248	0.3596	0.085*	
C18	0.4447 (2)	−0.1454 (2)	0.35699 (14)	0.0516 (4)	
C19	0.2880 (2)	−0.2002 (2)	0.32867 (15)	0.0548 (4)	
H19	0.2360	−0.2887	0.3400	0.066*	
C20	0.20833 (19)	−0.1236 (2)	0.28343 (16)	0.0552 (5)	
H20	0.1025	−0.1620	0.2630	0.066*	
C21	0.4639 (3)	−0.3534 (3)	0.4150 (2)	0.0850 (7)	
H21A	0.3928	−0.3336	0.4597	0.127*	
H21B	0.5405	−0.3892	0.4452	0.127*	
H21C	0.4103	−0.4315	0.3510	0.127*	
N1	0.68752 (13)	0.44938 (14)	0.17929 (9)	0.0359 (3)	
N2	0.56842 (13)	0.32172 (13)	0.16155 (10)	0.0366 (3)	
N3	0.24731 (14)	0.12398 (13)	0.11297 (10)	0.0388 (3)	
O1	0.90147 (18)	0.63540 (16)	0.39826 (10)	0.0687 (4)	
O2A	0.827 (4)	0.460 (2)	0.4645 (11)	0.074 (4)	0.40 (5)
O2B	0.763 (3)	0.4365 (10)	0.433 (2)	0.097 (5)	0.60 (5)
O3	0.20543 (16)	−0.13637 (13)	0.04358 (10)	0.0594 (3)	
O4	0.21707 (17)	0.26817 (15)	0.28507 (11)	0.0632 (4)	
O5	0.02109 (13)	0.02348 (18)	0.18811 (13)	0.0709 (4)	
O6	0.53520 (17)	−0.21420 (19)	0.39901 (13)	0.0750 (4)	
S1	0.17842 (4)	0.11051 (5)	0.21770 (4)	0.04718 (12)	
Cl1	0.76852 (5)	0.74258 (5)	0.18505 (4)	0.05352 (13)	

### Atomic displacement parameters (Å^2^)

**Table:**

	*U*^11^	*U*^22^	*U*^33^	*U*^12^	*U*^13^	*U*^23^
C1	0.0799 (15)	0.0598 (12)	0.0636 (13)	0.0125 (11)	−0.0014 (11)	0.0131 (10)
C2	0.0808 (15)	0.0679 (14)	0.0560 (12)	0.0081 (11)	0.0137 (11)	0.0050 (10)
C3	0.0460 (9)	0.0567 (11)	0.0697 (12)	0.0192 (8)	0.0124 (8)	0.0329 (9)
C4	0.0395 (8)	0.0446 (9)	0.0715 (12)	0.0145 (7)	0.0029 (8)	0.0193 (8)
C5	0.0288 (7)	0.0433 (8)	0.0569 (10)	0.0070 (6)	0.0096 (6)	0.0117 (7)
C6	0.0376 (7)	0.0283 (7)	0.0378 (7)	0.0020 (5)	0.0082 (6)	0.0115 (5)
C7	0.0369 (7)	0.0280 (6)	0.0366 (7)	0.0069 (5)	0.0079 (6)	0.0099 (5)
C8	0.0319 (7)	0.0262 (6)	0.0379 (7)	0.0070 (5)	0.0084 (5)	0.0088 (5)
C9	0.0305 (6)	0.0278 (6)	0.0434 (8)	0.0054 (5)	0.0076 (6)	0.0078 (5)
C10	0.0316 (7)	0.0413 (8)	0.0528 (9)	0.0101 (6)	0.0055 (6)	0.0106 (7)
C11	0.0443 (9)	0.0453 (9)	0.0557 (10)	0.0208 (7)	0.0054 (7)	0.0159 (7)
C12	0.0506 (9)	0.0329 (7)	0.0484 (9)	0.0156 (6)	0.0094 (7)	0.0157 (6)
C13	0.0380 (8)	0.0296 (7)	0.0484 (9)	0.0052 (6)	−0.0055 (6)	0.0063 (6)
C14	0.0582 (10)	0.0387 (8)	0.0445 (9)	0.0178 (7)	0.0012 (7)	0.0072 (7)
C15	0.0344 (7)	0.0408 (8)	0.0494 (9)	0.0031 (6)	0.0109 (6)	0.0155 (7)
C16	0.0376 (9)	0.0613 (12)	0.0818 (14)	−0.0078 (8)	0.0013 (9)	0.0393 (10)
C17	0.0359 (9)	0.0798 (15)	0.0981 (16)	−0.0058 (9)	−0.0025 (9)	0.0521 (13)
C18	0.0457 (9)	0.0578 (11)	0.0535 (10)	0.0098 (8)	0.0101 (7)	0.0253 (8)
C19	0.0460 (9)	0.0487 (10)	0.0719 (12)	0.0050 (7)	0.0163 (8)	0.0282 (9)
C20	0.0339 (8)	0.0500 (10)	0.0798 (13)	0.0009 (7)	0.0107 (8)	0.0277 (9)
C21	0.0747 (15)	0.0906 (18)	0.117 (2)	0.0263 (13)	0.0257 (14)	0.0693 (16)
N1	0.0290 (6)	0.0305 (6)	0.0447 (7)	0.0029 (4)	0.0062 (5)	0.0124 (5)
N2	0.0291 (6)	0.0274 (6)	0.0511 (7)	0.0045 (4)	0.0076 (5)	0.0130 (5)
N3	0.0328 (6)	0.0280 (6)	0.0501 (7)	0.0027 (5)	0.0085 (5)	0.0102 (5)
O1	0.0901 (11)	0.0518 (8)	0.0469 (7)	−0.0014 (7)	0.0122 (7)	0.0092 (6)
O2A	0.090 (8)	0.069 (5)	0.079 (5)	0.023 (5)	0.029 (5)	0.044 (3)
O2B	0.106 (9)	0.078 (3)	0.117 (9)	0.013 (4)	0.069 (8)	0.041 (4)
O3	0.0734 (9)	0.0294 (6)	0.0623 (8)	0.0020 (5)	−0.0003 (6)	0.0112 (5)
O4	0.0792 (9)	0.0511 (7)	0.0686 (8)	0.0254 (7)	0.0410 (7)	0.0198 (6)
O5	0.0303 (6)	0.0794 (10)	0.1197 (12)	0.0092 (6)	0.0205 (7)	0.0597 (9)
O6	0.0554 (8)	0.0882 (11)	0.0985 (12)	0.0154 (7)	0.0105 (8)	0.0603 (10)
S1	0.03440 (19)	0.0441 (2)	0.0676 (3)	0.00900 (16)	0.02084 (18)	0.02352 (19)
Cl1	0.0495 (2)	0.0355 (2)	0.0679 (3)	−0.00609 (16)	0.00440 (19)	0.02249 (18)

### Geometric parameters (Å, º)

**Table:**

C1—C2	1.475 (3)	C11—H11	0.9300
C1—H1A	0.9600	C12—H12	0.9300
C1—H1B	0.9600	C13—O3	1.2116 (19)
C1—H1C	0.9600	C13—N3	1.3996 (19)
C2—O1	1.464 (2)	C13—C14	1.488 (2)
C2—H2A	0.9700	C14—H14A	0.9600
C2—H2B	0.9700	C14—H14B	0.9600
C3—O2B	1.193 (6)	C14—H14C	0.9600
C3—O2A	1.233 (10)	C15—C20	1.377 (2)
C3—O1	1.305 (2)	C15—C16	1.387 (2)
C3—C4	1.498 (3)	C15—S1	1.7446 (18)
C4—C5	1.510 (3)	C16—C17	1.370 (3)
C4—H4A	0.9700	C16—H16	0.9300
C4—H4B	0.9700	C17—C18	1.389 (3)
C5—N1	1.4680 (19)	C17—H17	0.9300
C5—H5A	0.9700	C18—O6	1.357 (2)
C5—H5B	0.9700	C18—C19	1.375 (2)
C6—N1	1.3413 (19)	C19—C20	1.377 (3)
C6—C7	1.392 (2)	C19—H19	0.9300
C6—Cl1	1.6959 (14)	C20—H20	0.9300
C7—C12	1.410 (2)	C21—O6	1.423 (3)
C7—C8	1.4194 (19)	C21—H21A	0.9600
C8—N2	1.3464 (18)	C21—H21B	0.9600
C8—C9	1.4168 (18)	C21—H21C	0.9600
C9—C10	1.367 (2)	N1—N2	1.3602 (15)
C9—N3	1.4412 (18)	N3—S1	1.6932 (14)
C10—C11	1.416 (2)	O4—S1	1.4292 (13)
C10—H10	0.9300	O5—S1	1.4260 (13)
C11—C12	1.367 (2)		
			
C2—C1—H1A	109.5	C7—C12—H12	121.2
C2—C1—H1B	109.5	O3—C13—N3	120.00 (16)
H1A—C1—H1B	109.5	O3—C13—C14	123.63 (15)
C2—C1—H1C	109.5	N3—C13—C14	116.37 (13)
H1A—C1—H1C	109.5	C13—C14—H14A	109.5
H1B—C1—H1C	109.5	C13—C14—H14B	109.5
O1—C2—C1	108.42 (19)	H14A—C14—H14B	109.5
O1—C2—H2A	110.0	C13—C14—H14C	109.5
C1—C2—H2A	110.0	H14A—C14—H14C	109.5
O1—C2—H2B	110.0	H14B—C14—H14C	109.5
C1—C2—H2B	110.0	C20—C15—C16	119.85 (17)
H2A—C2—H2B	108.4	C20—C15—S1	119.72 (13)
O2B—C3—O2A	30.9 (5)	C16—C15—S1	120.34 (13)
O2B—C3—O1	126.8 (4)	C17—C16—C15	119.22 (16)
O2A—C3—O1	116.2 (10)	C17—C16—H16	120.4
O2B—C3—C4	119.1 (8)	C15—C16—H16	120.4
O2A—C3—C4	128.4 (6)	C16—C17—C18	120.96 (17)
O1—C3—C4	112.24 (16)	C16—C17—H17	119.5
C3—C4—C5	114.43 (15)	C18—C17—H17	119.5
C3—C4—H4A	108.7	O6—C18—C19	124.74 (17)
C5—C4—H4A	108.7	O6—C18—C17	115.74 (16)
C3—C4—H4B	108.7	C19—C18—C17	119.50 (18)
C5—C4—H4B	108.7	C18—C19—C20	119.67 (16)
H4A—C4—H4B	107.6	C18—C19—H19	120.2
N1—C5—C4	111.86 (13)	C20—C19—H19	120.2
N1—C5—H5A	109.2	C19—C20—C15	120.77 (15)
C4—C5—H5A	109.2	C19—C20—H20	119.6
N1—C5—H5B	109.2	C15—C20—H20	119.6
C4—C5—H5B	109.2	O6—C21—H21A	109.5
H5A—C5—H5B	107.9	O6—C21—H21B	109.5
N1—C6—C7	108.02 (12)	H21A—C21—H21B	109.5
N1—C6—Cl1	122.44 (11)	O6—C21—H21C	109.5
C7—C6—Cl1	129.55 (12)	H21A—C21—H21C	109.5
C6—C7—C12	135.94 (14)	H21B—C21—H21C	109.5
C6—C7—C8	102.85 (13)	C6—N1—N2	112.93 (12)
C12—C7—C8	121.18 (13)	C6—N1—C5	128.36 (12)
N2—C8—C9	127.74 (13)	N2—N1—C5	118.31 (12)
N2—C8—C7	112.71 (12)	C8—N2—N1	103.47 (11)
C9—C8—C7	119.54 (13)	C13—N3—C9	123.30 (13)
C10—C9—C8	118.45 (13)	C13—N3—S1	120.09 (11)
C10—C9—N3	121.44 (13)	C9—N3—S1	116.51 (10)
C8—C9—N3	120.08 (13)	C3—O1—C2	118.52 (17)
C9—C10—C11	121.33 (14)	C18—O6—C21	118.14 (16)
C9—C10—H10	119.3	O5—S1—O4	118.40 (9)
C11—C10—H10	119.3	O5—S1—N3	109.15 (8)
C12—C11—C10	121.85 (15)	O4—S1—N3	103.71 (7)
C12—C11—H11	119.1	O5—S1—C15	109.27 (8)
C10—C11—H11	119.1	O4—S1—C15	109.37 (9)
C11—C12—C7	117.60 (14)	N3—S1—C15	106.18 (7)
C11—C12—H12	121.2		
			
O2B—C3—C4—C5	126 (2)	C7—C6—N1—C5	171.20 (14)
O2A—C3—C4—C5	161.7 (19)	Cl1—C6—N1—C5	−8.9 (2)
O1—C3—C4—C5	−39.5 (2)	C4—C5—N1—C6	130.76 (16)
C3—C4—C5—N1	−59.63 (19)	C4—C5—N1—N2	−57.11 (18)
N1—C6—C7—C12	−176.92 (16)	C9—C8—N2—N1	179.12 (14)
Cl1—C6—C7—C12	3.2 (3)	C7—C8—N2—N1	0.16 (16)
N1—C6—C7—C8	1.25 (15)	C6—N1—N2—C8	0.70 (16)
Cl1—C6—C7—C8	−178.68 (12)	C5—N1—N2—C8	−172.61 (13)
C6—C7—C8—N2	−0.88 (16)	O3—C13—N3—C9	−175.04 (14)
C12—C7—C8—N2	177.63 (13)	C14—C13—N3—C9	4.8 (2)
C6—C7—C8—C9	−179.94 (13)	O3—C13—N3—S1	1.2 (2)
C12—C7—C8—C9	−1.4 (2)	C14—C13—N3—S1	−179.00 (11)
N2—C8—C9—C10	−176.42 (14)	C10—C9—N3—C13	97.10 (18)
C7—C8—C9—C10	2.5 (2)	C8—C9—N3—C13	−85.05 (18)
N2—C8—C9—N3	5.7 (2)	C10—C9—N3—S1	−79.22 (17)
C7—C8—C9—N3	−175.44 (12)	C8—C9—N3—S1	98.63 (14)
C8—C9—C10—C11	−1.6 (2)	O2B—C3—O1—C2	18 (2)
N3—C9—C10—C11	176.25 (14)	O2A—C3—O1—C2	−16.5 (14)
C9—C10—C11—C12	−0.4 (3)	C4—C3—O1—C2	−178.05 (17)
C10—C11—C12—C7	1.5 (2)	C1—C2—O1—C3	121.8 (2)
C6—C7—C12—C11	177.37 (16)	C19—C18—O6—C21	0.2 (3)
C8—C7—C12—C11	−0.5 (2)	C17—C18—O6—C21	−178.6 (2)
C20—C15—C16—C17	1.0 (3)	C13—N3—S1—O5	−63.19 (14)
S1—C15—C16—C17	177.49 (17)	C9—N3—S1—O5	113.25 (12)
C15—C16—C17—C18	−1.4 (3)	C13—N3—S1—O4	169.72 (12)
C16—C17—C18—O6	179.3 (2)	C9—N3—S1—O4	−13.83 (13)
C16—C17—C18—C19	0.5 (3)	C13—N3—S1—C15	54.49 (13)
O6—C18—C19—C20	−177.76 (18)	C9—N3—S1—C15	−129.06 (11)
C17—C18—C19—C20	0.9 (3)	C20—C15—S1—O5	−6.70 (18)
C18—C19—C20—C15	−1.4 (3)	C16—C15—S1—O5	176.77 (16)
C16—C15—C20—C19	0.4 (3)	C20—C15—S1—O4	124.38 (15)
S1—C15—C20—C19	−176.12 (15)	C16—C15—S1—O4	−52.16 (17)
C7—C6—N1—N2	−1.29 (17)	C20—C15—S1—N3	−124.30 (15)
Cl1—C6—N1—N2	178.64 (10)	C16—C15—S1—N3	59.16 (17)

### Hydrogen-bond geometry (Å, º)

**Table:**

*D*—H···*A*	*D*—H	H···*A*	*D*···*A*	*D*—H···*A*
C2—H2*A*···O4^i^	0.97	2.58	3.438 (3)	148
C21—H21*A*···O2*B*^ii^	0.96	2.44	3.273 (7)	144
C21—H21*A*···O2*A*^ii^	0.96	2.60	3.43 (2)	145
C2—H2*B*···O6^iii^	0.97	2.71	3.510 (3)	140
C14—H14*C*···N2^iv^	0.96	2.55	3.501 (2)	173
C14—H14*B*···O5^v^	0.96	2.45	3.348 (2)	155

Symmetry codes: (i) −*x*+1, −*y*+1, −*z*+1; (ii) −*x*+1, −*y*, −*z*+1; (iii) *x*, *y*+1, *z*; (iv) −*x*+1, −*y*, −*z*; (v) −*x*, −*y*, −*z*.
